# Interaction of Isoflavones and Endophyte-Infected Tall Fescue Seed Extract on Vasoactivity of Bovine Mesenteric Vasculature

**DOI:** 10.3389/fnut.2015.00032

**Published:** 2015-10-19

**Authors:** Yang Jia, David L. Harmon, Michael D. Flythe, James L. Klotz

**Affiliations:** ^1^Department of Animal and Food Sciences, University of Kentucky, Lexington, KY, USA; ^2^Forage-Animal Production Research Unit, Agricultural Research Service, United States Department of Agriculture, Lexington, KY, USA

**Keywords:** ergot alkaloids, isoflavones, mesenteric vasculature, vasoconstriction

## Abstract

It was hypothesized that isoflavones may attenuate ergot alkaloid-induced vasoconstriction and possibly alleviate diminished contractility of vasculature after exposure to ergot alkaloids. The objective of this study was to determine if prior incubation of bovine mesenteric vasculature with the isoflavones formononetin (*F*), biochanin A (*B*), or an ergovaline-containing tall fescue seed extract (EXT) and their combinations affect ergotamine (ERT)-induced contractility. Multiple segments of mesenteric artery and vein supporting the ileal flange of the small intestine were collected from Angus heifers at slaughter (*n* = 5, bodyweight = 639 ± 39 kg). Duplicates of each vessel type were incubated in tissue culture flasks at 37°C with a 50-mL volume of Krebs–Henseleit buffer containing: only buffer (control); or 1 × 10^−6^ M EXT; *F*; or *B*; and combinations of 1 × 10^−6^ M EXT + *F*; 1 × 10^−6^ M EXT + *B*; 1 × 10^−6^ M *F* + *B*; or 1 × 10^−6^ M EXT + *F* + *B*. After incubation for 2 h, sections were mounted in a multimyograph chamber. The ERT dose responses were normalized to 0.12 M KCl. Pretreatment with *F*, *B*, and *F* + *B* without EXT resulted in similar contractile responses to ERT in mesenteric artery and all incubations containing EXT resulted in a complete loss of vasoactivity to ERT. In mesenteric artery pretreated with EXT, treatments that contained *B* had higher contractile responses (*P* < 0.05) at ERT concentrations of 1 × 10^−7^ and 5 × 10^−7^ M. Also, treatments containing *B* tended (*P* < 0.1) to have greater responses than treatments without *B* at ERT concentrations of 1 × 10^−6^, 5 × 10^−6^, and 5 × 10^−5^ M. In mesenteric vein pretreated with EXT, treatments containing *F* had greater contractile responses to ERT at 1 × 10^−5^, 5 × 10^−5^, and 1 × 10^−4^ M (*P* < 0.05). These data indicated that *F* and *B* at 1 × 10^−6^ M and their combination did not impact the overall contractile response to ERT in mesenteric vasculature. However, *F* and *B* may offset some of the vasoconstriction caused by prior exposure to ergot alkaloids.

## Introduction

As a symbiotic endophyte of tall fescue (*Lolium arundinaceum*) (1–4), *Epichloë coenophiala* produces a variety of ergot alkaloids (5, 6), which have been identified as causative agents of vasoconstriction and symptoms of fescue toxicosis in grazing animals (7, 8). Ergovaline and ergotamine commonly draw more attention of researchers. Lyons et al. (5) reported that ergovaline was the predominant (84–97%) alkaloid of all the five detected ergopeptine alkaloids from tall fescue (*Lolium arundinaceum*) pasture. Numerous studies have reported that ergot alkaloids (ergovaline and ergotamine) induce vasoconstriction in peripheral blood vessel models such as the caudal artery (9, 10), dorsal pedal vein (11), and lateral saphenous vein (12), and also core blood vessel models like the right ruminal artery and vein (13), bovine uterine and umbilical arteries (14), and mesenteric artery and vein (15). Consistent with findings in the bovine lateral saphenous vein (16, 17), Egert et al. (15) demonstrated that previous dietary exposure to ergot alkaloids reduced the vasoactivity in bovine mesenteric vasculature.

Isoflavones are almost exclusively found in the legume (*Leguminosae/Fabaceae*) family, such as soybean, chickpeas, and red clover (18, 19). A variety of isoflavones, such as genistein, daidzein, biochanin A (*B*), and formononetin (*F*), have been shown to elicit different beneficial effects on humans in many different ways, for example, improvements to the cardiovascular system, osteoporosis, anti-breast, and prostate cancer (20–23). Substantial evidence has been published on the vasodilative effects of isoflavones and their metabolites in different vessel types in humans (24, 25) and rats (26–29). However, based on our knowledge, no studies have investigated the vasodilative effects of isoflavones on bovine vessels.

Ergot alkaloids share some structural similarities with biogenic amines (i.e., dopamine, epinephrine, norepinephrine, serotonin) and thus can cause vasoconstriction by binding biogenic amine receptors (30) found throughout the body. On the other hand, isoflavones have estrogenic activities and could cause endothelium-dependent or -independent vasorelaxation. Although the precise mechanisms behind the vascular bioactivity of both ergot alkaloids and isoflavones have not been fully defined, current knowledge suggests that they are triggered by different mechanisms. Nevala et al. (31) reported that isoflavones relax noradrenaline precontracted rat mesenteric arteries. Likewise, Egert et al. (15) demonstrated that ergot alkaloids were vasoconstrictive in bovine mesenteric vasculature, whereas dietary exposure to ergot alkaloids decreased the contractility of mesenteric vasculature. Thus, it was hypothesized that isoflavones may attenuate ergot alkaloid-induced vasoconstriction and possibly alleviate the diminished contractility of mesenteric vasculature after preliminary exposure to ergot alkaloids. The objective of this study was to determine if an incubation of bovine mesenteric vasculature with *F*, *B*, or ergovaline-containing tall fescue seed extract (EXT) and their combinations affect ergotamine (ERT)-induced contractility.

## Materials and Methods

No live animals were involved this study, so approval from the University of Kentucky Animal Care and Use Committee was not required.

### Animals and Tissue Collection

Five Angus heifers (Bodyweight = 639 ± 39 kg) were slaughtered and tissues were collected at the University of Kentucky abattoir. As originally described by Klotz and Barnes (32), the gastrointestinal tract was removed from the carcass, and the cecum, ileocecal fold, and the ileal flange were identified as landmarks. Within the mesentery supporting the ileal flange, multiple branches of the mesenteric artery and vein bundles were dissected and submerged in oxygenated Krebs–Henseleit buffer (95% O_2_/5% CO_2_; pH = 7.4; 11.1 mM d-glucose; 1.2 mM MgSO_4_; 1.2 mM KH_2_PO_4_; 4.7 mM KCl; 118.1 mM NaCl; 3.4 mM CaCl_2_; 24.9 mM NaHCO_3_; Sigma Chemical Co., St. Louis, MO, USA) for transport to the laboratory. Samples were stored on ice until cleaned. At the time of cleaning, surrounding fat and connective tissues were carefully removed, and mesentery artery and vein were separated under a magnifying lamp (2.5 to 5.0× magnification). Cleaned vessels were sliced into 2-mm segments and examined under a dissecting scope (Semi 2000-C, Carl Zeiss Inc., Oberkochen, Germany) at 12.5× magnification to ensure the usability of the vessels. Cross-sections with abnormalities (branches, valves, or structural damage) were replaced with structurally integral ones.

### Pre-Myograph Incubations

A tall fescue seed extract was prepared as described by Foote et al. (33) to contain a 1 × 10^−6^ M working concentration of ergovaline. Duplicates of each vessel type (from each animal) were incubated in tissue culture flasks with a 50-mL volume of Krebs–Henseleit buffer containing: only buffer (control); 1 × 10^−6^ M EXT; 1 × 10^−6^ M *F* (≥99.0%; 47752-5MG-*F*; Sigma Chemical Co., St. Louis, MO, USA); or 1 × 10^−6^ M *B* (D2016; Sigma Chemical Co., St. Louis, MO, USA); and combinations of 1 × 10^−6^ M EXT + *F*; 1 × 10^−6^ M EXT + *B*; 1 × 10^−6^ M *F* + *B*; or 1 × 10^−6^ M EXT + *F* + *B*. All buffer solutions were prewarmed for 30 min in a CO_2_ incubator (95% O_2_/5% CO_2_; 37°C; Nu-8500, NUAIRE, Inc., Plymouth, MN, USA) prior to blood vessel addition. Duplicate blood vessel segments were randomly placed into each treatment flask and incubated in the same conditions for 2 h. Immediately after the 2-h incubation, dimensional measurements of cross-sections were recorded only for mesentery artery using Axiovision (version 20, Carl Zeiss, Inc.).

### Experimental Myograph Protocol

Following the 2-h incubations, an ERT concentration response experiment was conducted using the procedures described by Klotz and Barnes (32). ERT (ergotamine d-tartrate; 97%; 45510; Aldrich, Milwaukee, WI, USA) standards were prepared by diluting a stock solution (0.0201 M) with dimethyl sulfoxide to working concentrations that resulted in a concentration range of 5 × 10^−9^ to 1 × 10^−4^ M in the myograph chamber (contained 5 mL of Krebs–Henseleit buffer).

Artery and vein cross-sections were mounted on the myograph (Multichamber myograph; DMT 610M, Danish Myo Technology, Atlanta, GA, USA) by inserting the supports through the lumen in individual myograph chambers containing 5 mL modified Krebs–Henseleit buffer and continuously gassed (95% O_2_/5% CO_2_; pH = 7.4; 37°C). The incubation buffer was modified from transport Krebs–Henseleit buffer by adding desipramine (3 × 10^−5^ M; D3900; Sigma Chemical Co.) to inhibit the reuptake mechanisms of biogenic amines and propranolol (1 × 10^−6^ M; P0844; Sigma Chemical Co.) to block the non-specific binding of ERT to β-adrenergic receptors. A 90-min equilibration period with buffer replacement occurring every 15 min was completed based on the conditions above to achieve a stable resting tension of approximately 1 g. Following the equilibration period, the blood vessels were exposed to 120 mM KCl for 15 min to evaluate tissue viability and to normalize treatment data. Following the KCl addition, the incubation buffer was replaced every 15 min until vessel tension returned to the 1 g baseline. Once the vessel returned to baseline, addition of ERT standards was initiated in order to increase the concentration. ERT additions were added in 15-min intervals consisting of a 9-min-incubation period, two 2.5-min buffer washes, and a third, final buffer replacement that was followed by 1-min recovery before the next ERT addition. This 15 min cycle was repeated for the rest of the nine remaining ERT additions. Following the 1-min recovery after the final ERT addition, vessels were again exposed to 120 mM KCl to confirm the vessel viability at the end of the experiment.

### Data Collection

The isometric contractions of the different preincubated mesenteric vessels to KCl and ERT additions were digitized and recorded as grams of tension using a PowerLab/8sp and Chart software (version 7.3, ADInstruments, Colorado Springs, Co.). Baseline tension was measured immediately before the addition of 120 mM KCl. For all contractile response data, the maximum observed tension (in grams) in the 9 min-incubation period was recorded as the contractile response. Contractile response data were corrected for baseline tension and normalized as a percentage of the reference compound KCl induced maximum contractile response. The differences of tissue response due to the variations of vessel size and across individual animal were minimized by this data normalization. The contractile response to ERT was determined and presented as percentage means ± SEM. A measurement of potency or the half-maximal effective concentration (EC_50_) was calculated using GraphPad Prism (version 5; GraphPad Software Inc., La Jolla, CA, USA) using a non-linear regression with fixed slope. The sigmoidal concentration response curves of pretreated mesentery artery and vein to ERT was plotted by using a three-parameter equation:

$$y=\text{bottom}+\left(\frac{\text{top-bottom}}{\left[1+10^{\left(\text{log}{\text{EC}}_{50}-x\right)}\right]}\right),$$

where *y* represents contractile response, and *x* denotes the agonist concentration, top and bottom are the plateaus of contractile response as percentage of 120 mM KCl maximum response. The EC_50_ is the molar concentration of ERT inducing 50% of the KCl maximum response.

### Statistics

All data were analyzed using the MIXED model of SAS (SAS 9.4, SAS Inst. Inc., Cary, NC, USA). Contractile response data of mesentery artery and vein were analyzed separately as two datasets for treatments with and without EXT. Data for contractile response (within each ERT concentration), KCl maximal response, inside and outside diameters for mesenteric artery (these data could not be obtained for mesenteric vein samples due to the pliable nature of this vessel) were analyzed as a completely randomized design with a factorial treatment arrangement. The fixed effects included the effects of *F*, *B*, and the interaction of *F* × *B* in the presence or absence of EXT. Due to the shape of the response curve, EC_50_ data were analyzed only in treatments without EXT from mesentery artery using a completely randomized design with treatment as fixed variable. For all data, pair-wise comparisons of least square means (±SEM) were only performed if the probability of a greater *F*-statistic from the analysis of variance was significant for the tested effect and interaction. Mean separation was performed with the LSD feature of SAS. Differences are denoted as significant at *P* < 0.05, unless specifically reported otherwise.

## Results

**Figure 1:**
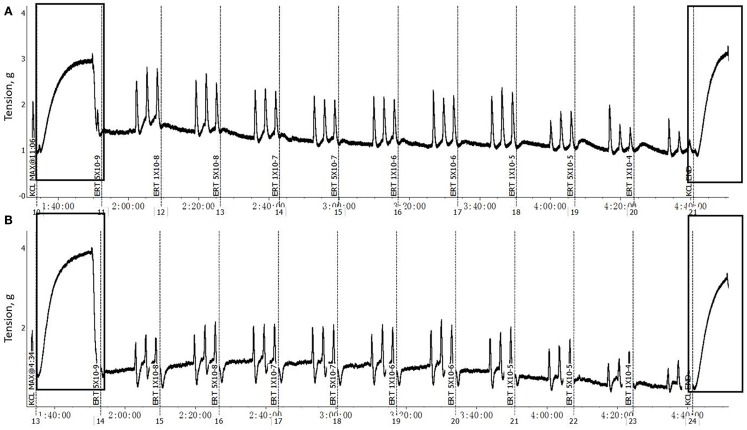
**Example of a typical response of mesenteric artery (A) and vein (***B***) cross-sections, after pretreated with 1 **×** 10^−6^ M EXT and 1 **×** 10^−6^ M *F* and 1 **×** 10^−6^ M *B* (EXT **+** *F* **+** *B*), to increasing concentrations of ERT (5 **×** 10^−9^ to 1 **×** 10^−4^ M)**. The rectangles highlighted regions are the initial and end KCl (0.12 M) additions.

The *F* and *B* incubation pretreatment did not impact (*P* > 0.05) the maximum contractile response of mesenteric artery or mesenteric vein to 120 mM KCl either without EXT (Table 1) or with EXT (Table 2). The pretreatments with EXT did not compromise the vessel viability, which was indicated by the ending response to KCl for either the artery or vein (Figure 1). In mesenteric artery, the inside and outside diameters after the 2 h incubation were not affected (*P* > 0.05) by *F* or *B* in both treatment groups with (Table 2) or without EXT (Table 1). However, there was a tendency (*P* = 0.07) for *F* treated vessels to have a larger inside diameter for those pretreated with EXT (Table 2), and a smaller outside diameter (*P* = 0.09) for those not pretreated with EXT (Table 1). Interestingly, mesenteric artery pretreated with *B* tended (*P* = 0.06) to have a smaller inside diameter when incubated with EXT (Table 2).

**Table 1:** **Inside diameter, outside diameter, and SEM of mesenteric artery, and the mean KCl maximum response of mesenteric artery and vein to pretreatment without tall fescue extract: only Krebs–Henseleit buffer (control); 1 **×** 10^−^^6^ M treatments of formononetin (*F*), biochanin A (*B*), and combination of *F* and *B* (*F* **+** *B*)**.

Item	Control	*F*	*B*	*F* **+** *B*	SEM	*P*-value		
						*F*	*B*	*F* × *B*
**Mesenteric artery**								
KCl maximum response (g)	4.36	4.42	4.22	3.66	0.46	0.58	0.33	0.50
Inside diameter (mm)	0.81	0.79	0.86	0.77	0.06	0.38	0.77	0.61
Outside diameter (mm)	1.72	1.64	1.75	1.57	0.07	0.09	0.74	0.47
**Mesenteric vein^a^**								
KCl maximum response (g)	1.70	1.81	1.89	2.22	0.21	0.31	0.18	0.62

*^a^Due to the elasticity of the mesenteric vein, measurements of vascular dimensions were not possible*.

**Table 2:** **Inside diameter, outside diameter, and SEM of mesenteric artery, and the mean KCl maximum response of mesenteric artery and vein to pretreatment with tall fescue seed extract: 1 **×** 10^−^^6^ M ergovaline-containing tall fescue seed extract (EXT); combinations of 1 **×** 10^−^^6^ M EXT and formononetin (*F*), EXT and biochanin A (*B*), and the combination of EXT **+** *F* **+** *B***.

Item	EXT	EXT + *F*	EXT + *B*	EXT + *F* **+** *B*	SEM	*P*-value		
						*F*	*B*	*F* × *B*
**Mesenteric artery**								
KCl maximum response (g)	3.43	2.68	3.08	2.84	0.37	0.19	0.80	0.50
Inside diameter (mm)	0.72	0.78	0.60	0.72	0.05	0.07	0.06	0.50
Outside diameter (mm)	1.64	1.57	1.51	1.59	0.07	0.92	0.43	0.29
**Mesenteric vein**^a^								
KCl maximum response (g)	1.90	2.20	2.09	2.49	0.21	0.11	0.26	0.81

*^a^Due to the elasticity of the mesenteric vein, measurements of vascular dimensions were not possible*.

**Figure 2:**
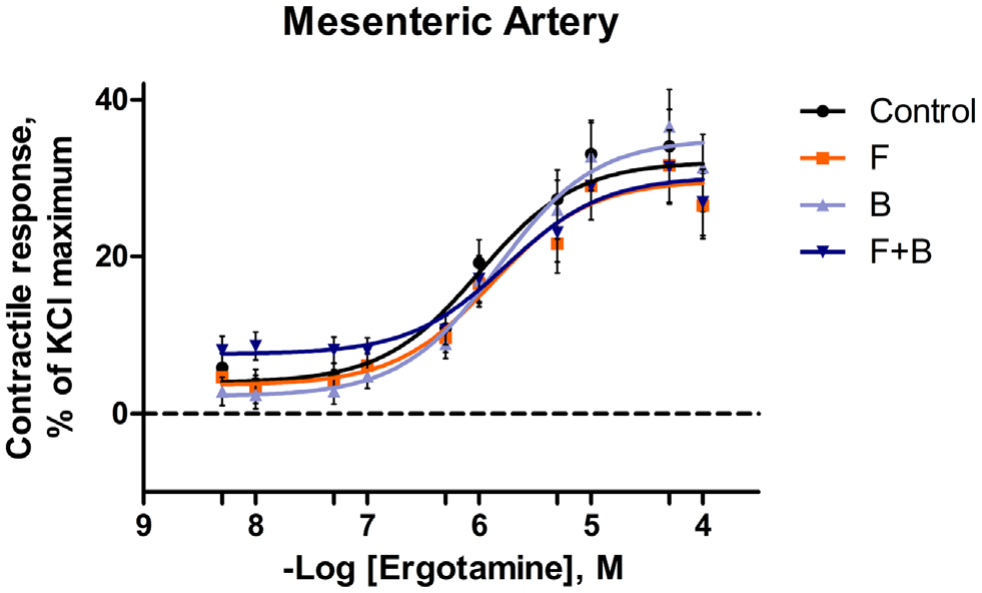
**Mean contractile response, as % KCl maximum of mesenteric artery to increasing concentrations of ergotamine for pretreatments without tall fescue seed extract: only Krebs–Henseleit buffer (control); 1 **×** 10^−6^ M formononetin (*F*); or 1 **×** 10^−6^ M biochanin A (*B*); and combination of 1 **×** 10^−6^ M *F* and 1 **×** 10^−6^ M *B* (*F* **+** *B*)**. The regression lines were plotted for each treatment using a non-linear regression with fixed slope, and the sigmoidal concentration response curves were calculated by the following equation: $y=\text{bottom}+\left[\text{(top-bottom)/(1}+{\text{10}}^{\left(\text{log}EC_{50}-x\right)}\text{)}\right],$ where top and bottom are the plateaus of contractile response as percentage of 120 mM KCl maximum response. EC_50_ is the molar concentration of ergotamine inducing 50% of the KCl maximum response.

**Figure 3:**
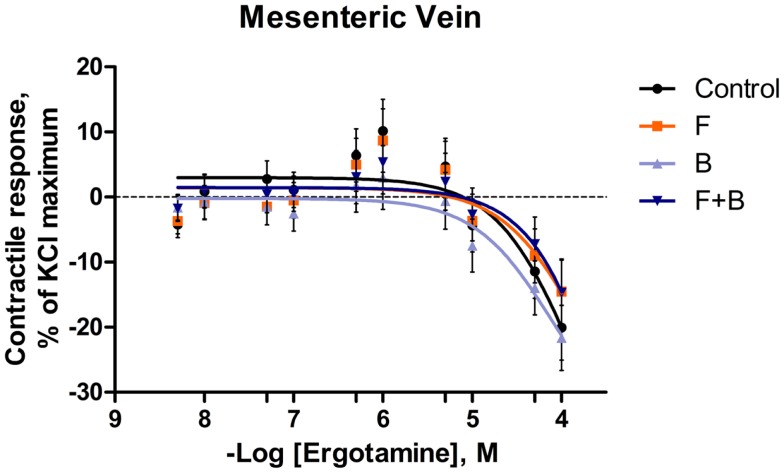
**Mean contractile response, as % KCl maximum of mesenteric vein to increasing concentrations of ergotamine for pretreatments without tall fescue seed extract: only Krebs–Henseleit buffer (control); 1 **×** 10^−6^ M formononetin (*F*); or 1 **×** 10^−6^ M biochanin A (*B*); and combination of 1 **×** 10^−6^ M *F* and 1 **×** 10^−6^ M *B* (*F* **+** *B*)**. The regression lines were plotted for each treatment using a non-linear regression with fixed slope, and the sigmoidal concentration response curves were calculated by the following equation: $y=\text{bottom}+\left[\text{(top-bottom)/(1}+{\text{10}}^{\left(\text{log}EC_{50}-x\right)}\text{)}\right],$ where top and bottom are the plateaus of contractile response as percentage of 120 mM KCl maximum response. EC_50_ is the molar concentration of ergotamine inducing 50% of the KCl maximum response.

In the mesenteric artery, ERT induced similar contractile responses in all treatments (Figure 2) with −log^EC50^ values (5.99 ± 0.14, 5.80 ± 0.14, 5.88 ± 0.14, 5.74 ± 0.14 M, respectively) that did not differ with each other (*P* = 0.63). Within each ERT concentration, tendencies for *F* × *B* interactions in mesenteric artery were observed at ERT 5 × 10^−9^, 1 × 10^−8^, 5 × 10^−8^ M (*P* = 0.09, *P* = 0.07, *P* = 0.08, respectively; Table 3) for treatments without EXT. Contractile responses of *F* + *B* treated mesentery artery were greater than *B* at ERT concentrations 1 × 10^−8^ and 5 × 10^−8^ M (*P* < 0.05). In mesenteric vein, ERT-induced contractile responses reached the maximum response at 1 × 10^−6^ M for Control, *B*, *F*, *F* + *B* treatments (Figure 3), and then relaxed to negative response values with increases in ERT concentration.

**Table 3:** **The analysis of variance and *P*-values of main effect of formononetin (*F*), biochanin A (*B*), and the interaction of formononetin and biochanin A (*F* **×** *B*) for pretreatments without tall fescue extract: only Krebs–Henseleit buffer; 1 **×** 10^−^^6^ M *F*, *B*, and combination of *F* and *B* on every ergotamine concentration**.

Ergotamine concentration (M)	*P*-value		
	*F*	*B*	*F* **×** *B*
**Mesenteric artery**			
5 × 10^−9^	0.23	0.91	0.09
1 × 10^−8^	0.14	0.27	0.07
5 × 10^−8^	0.20	0.51	0.08
1 × 10^−7^	0.29	0.83	0.31
5 × 10^−7^	0.88	0.79	0.43
1 × 10^−6^	0.68	0.78	0.64
5 × 10^−6^	0.27	0.99	0.72
1 × 10^−5^	0.38	0.97	0.97
5 × 10^−5^	0.43	0.81	0.77
1 × 10^−4^	0.61	0.54	0.59
**Mesenteric vein**			
5 × 10^−9^	0.92	0.27	0.85
1 × 10^−8^	1.00	0.96	0.50
5 × 10^−8^	0.67	0.67	0.28
1 × 10^−7^	0.80	0.64	0.41
5 × 10^−7^	0.99	0.43	0.74
1 × 10^−6^	0.93	0.30	0.70
5 × 10^−6^	0.78	0.43	0.70
1 × 10^−5^	0.50	0.80	0.62
5 × 10^−5^	0.29	0.93	0.61
1 × 10^−4^	0.23	0.86	0.89

**Figure 4:**
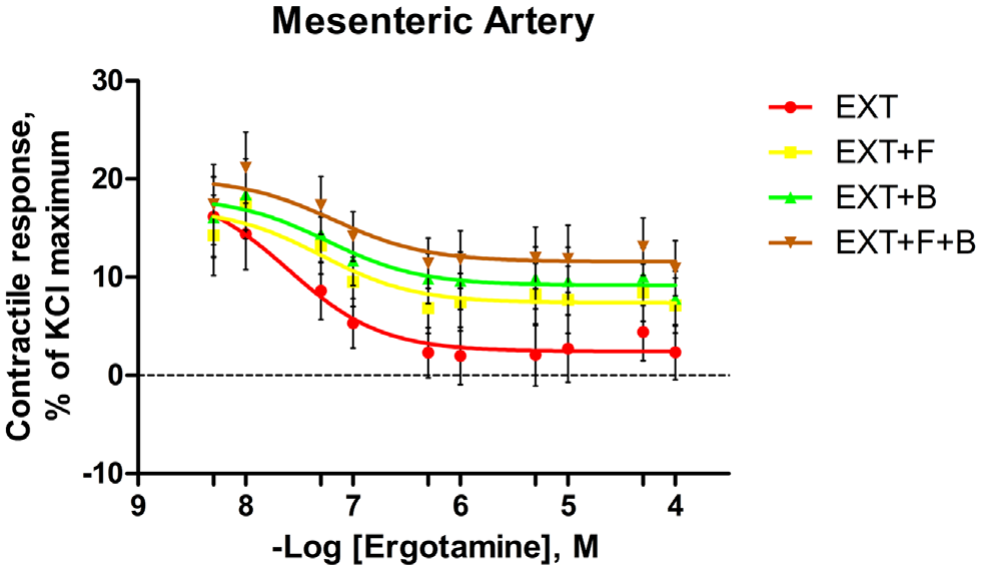
**Mean contractile response, as % KCl maximum of mesenteric artery to increasing concentrations of ergotamine for pretreatments with tall fescue seed extract: 1 **×** 10^−6^ M ergovaline-containing tall fescue seed extract (EXT); combinations of 1 **×** 10^−6^ M EXT and 1 **×** 10^−6^ M *F* (EXT **+** *F*); 1 **×** 10^−6^ M EXT and 1 **×** 10^−6^ M *B* (EXT **+** *B*); or 1 **×** 10^−6^ M EXT and 1 **×** 10^−6^ M *F* and 1 **×** 10^−6^ M *B* (EXT **+** *F* **+** *B*)**. The regression lines were plotted for each treatment using a non-linear regression with fixed slope, and the sigmoidal concentration response curves were calculated by the following equation: $y=\text{bottom}+\left[\text{(top-bottom)/(1}+{\text{10}}^{\left(\text{log}EC_{50}-x\right)}\text{)}\right],$ where top and bottom are the plateaus of contractile response as percentage of 120 mM KCl maximum response. EC_50_ is the molar concentration of ergotamine inducing 50% of the KCl maximum response.

**Figure 5:**
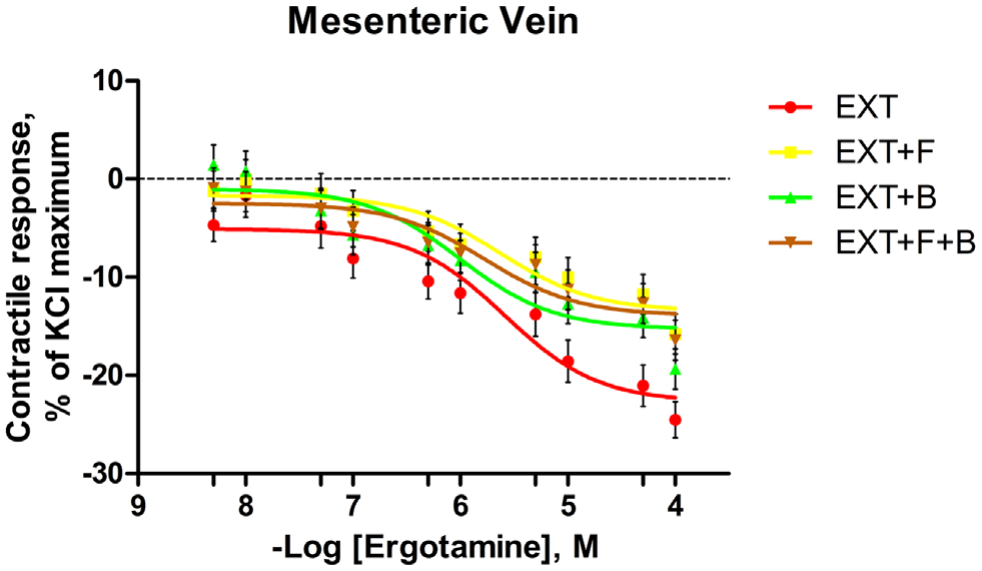
**Mean contractile response, as % KCl maximum of mesenteric vein to increasing concentrations of ergotamine for pretreatments with tall fescue seed extract: 1 **×** 10^−6^ M ergovaline-containing tall fescue seed extract (EXT); combinations of 1 **×** 10^−6^ M EXT and 1 **×** 10^−6^ M *F* (EXT **+** *F*); 1 **×** 10^−6^ M EXT and 1 **×** 10^−6^ M *B* (EXT **+** *B*); or 1 **×** 10^−6^ M EXT and 1 **×** 10^−6^ M *F* and 1 **×** 10^−6^ M *B* (EXT **+** *F* **+** *B*)**. The regression lines were plotted for each treatment using a non-linear regression with fixed slope, and the sigmoidal concentration response curves were calculated by the following equation: $y=\text{bottom}+\left[\text{(top-bottom)/(1}+{\text{10}}^{\left(\text{log}EC_{50}-x\right)}\text{)}\right],$ where top and bottom are the plateaus of contractile response as percentage of 120 mM KCl maximum response. EC_50_ is the molar concentration of ergotamine inducing 50% of the KCl maximum response.

For the blood vessels incubated with EXT, the contractile responses decreased as the concentration of ERT increased in both mesenteric artery (Figure 4) and mesenteric vein (Figure 5). In mesenteric artery, a main effect of *B* was observed at ERT concentrations of 1 × 10^−7^ and 5 × 10^−7^ M (Table 4), with treatments that contained *B* having higher contractile responses (*P* < 0.05). Also, treatments containing *B* tended (*P* < 0.1) to have greater responses than treatments without *B* at ERT concentrations of 1 × 10^−6^, 5 × 10^−6^, and 5 × 10^−5^ M (Table 4).

**Table 4:** **The analysis of variance and *P*-values of main effect of formononetin (*F*), biochanin A (*B*), and the interaction of formononetin and biochanin A (*F* **×** *B*) for pretreatments with tall fescue seed extract (EXT): 1 **×** 10^−^^6^ M (EXT); combinations of 1 **×** 10^−^^6^ M EXT and *F*, EXT and *B*, or 1 **×** 10^−^^6^ M EXT **+** *B* **+** *F* on every ergotamine concentration**.

Ergotamine concentration (M)	*P*-value		
	*F*	*B*	*F* **×** *B*
**Mesenteric artery**			
5 × 10^−9^	0.94	0.71	0.70
1 × 10^−8^	0.43	0.31	0.96
5 × 10^−8^	0.22	0.11	0.77
1 × 10^−7^	0.20	0.04	0.73
5 × 10^−7^	0.25	0.03	0.57
1 × 10^−6^	0.21	0.06	0.58
5 × 10^−6^	0.21	0.09	0.52
1 × 10^−5^	0.31	0.13	0.70
5 × 10^−5^	0.24	0.10	0.87
1 × 10^−4^	0.18	0.12	0.77
**Mesenteric vein**			
5 × 10^−9^	0.75	0.07	0.10
1 × 10^−8^	0.91	0.77	0.41
5 × 10^−8^	0.45	0.98	0.50
1 × 10^−7^	0.18	0.85	0.32
5 × 10^−7^	0.15	0.48	0.19
1 × 10^−6^	0.19	0.58	0.32
5 × 10^−6^	0.16	0.44	0.27
1 × 10^−5^	0.03	0.30	0.12
5 × 10^−5^	0.02	0.17	0.08
1 × 10^−4^	0.01	0.24	0.14

In the mesenteric vein, the contractile response of all treatments with EXT to ERT decreased and remained below zero from the second ERT addition (1 × 10^−8^ M) to the last addition at 1 × 10^−4^ M. Tendencies for main effects of *B* (*P* = 0.06) and *F* × *B* interaction (*P* = 0.09) were observed at ERT concentrations of 5 × 10^−9^ M, where EXT treatment had a lower contractile response than EXT + *B* (*P* < 0.05; Table 4). Treatments containing *F* had greater contractile responses to ERT at 1 × 10^−5^, 5 × 10^−5^, and 1 × 10^−4^ M in mesenteric vein (main effect of *F*; *P* < 0.05; Table 4). Within the 5 × 10^−5^ M ERT concentration, the contractile response of EXT was the lowest (*P* = 0.03).

## Discussion

This is the first study to investigate the interaction of ergot alkaloids and isoflavones on bovine mesenteric vasculature. Physiological effects of isoflavones and their metabolites on vasculature have been extensively studied on different vessel types in humans (24, 25) and rat models (26–29). The concentrations of isoflavones (1 × 10^−6^ M formononetin and biochanin A) used in the current experiment were based on a study that reported dose-dependent formononetin- and biochanin A-induced relaxations of rat-isolated thoracic aorta precontracted with phenylephrine (29). At 1 × 10^−6^ M, formononetin and biochanin A were capable of inducing about 35 and 25% relaxation, respectively. Since the current study was the first to investigate interactions of isoflavones and ergot alkaloids, our rationale was to pick an intermediate concentration (1 × 10^−6^ M) to avoid extreme scenarios and one that was equimolar with the concentration of ergovaline. The concentration of ergovaline-containing tall fescue seed extract that was chosen (1 × 10^−6^ M) is based on the findings of Egert et al. (15), where 1 × 10^−6^ M of ergovaline-containing extract was observed to induce contractile response (about 40% of KCl maximum) of bovine mesenteric artery and vein.

Several studies have demonstrated that cattle grazing endophyte-infected tall fescue had reduced contractile responses to 5-hydroxytryptamine (5-HT) and as well as ergot alkaloids in the lateral saphenous vein (16, 17). Recently, a study using mesenteric artery and vein from steers that had been ruminally dosed with endophyte-infected tall fescue seed observed a decreased or completely absent constrictive response to ergot alkaloids (15). In the current study, an *in vitro* incubation of bovine mesenteric vasculature in a medium containing EXT was used to achieve an ergot alkaloid pretreatment. The pretreatment of blood vessels with EXT (33) and isoflavones (*F* and *B*) were conducted at equimolar final concentrations of 1 × 10^−6^ M. It has been reported that *F* and *B* both induced vasorelaxation in phenylephrine-precontracted rat-isolated thoracic aorta at 1 × 10^−6^ M (29). This latter study identified both nitric oxide from endothelial nitric oxide synthase (NOS) and potassium efflux from endothelial cells as putative mechanisms of action. However, the 1 × 10^−6^ M ergovaline in the EXT may be considered a high dose compared to the physiological levels encountered by cattle grazing endophyte-infected tall fescue (34). Nevertheless, the viability of mesenteric artery and vein was not compromised by concentration of ergot alkaloids used in the current study, as evidenced by the fact that both artery and vein were responsive to the final KCl addition.

Studies have reported that many isoflavones and their metabolites can reduce the vasoconstriction induced by KCl in several different vessel types using rat models (28, 35). The contractile response to KCl of endothelium-denuded rat aortic rings was inhibited by pretreatment with genistein or daidzein at both 3 × 10^−5^ and 1 × 10^−4^ M for 30 min (36). However, in the same study, pretreatment with genistein or daidzein at 1 × 10^−5^ M did not relax the KCl-induced vasoconstriction. The similar dose-dependent inhibition of *F* (1 × 10^−5^, 3 × 10^−5^, and 1 × 10^−4^ M) on the contractile response to KCl was observed in rat mesenteric arteries without endothelium (37), where *F* again failed to inhibit KCl-initiated contraction at 1 × 10^−5^ M. However, in endothelium-intact rat aortic rings, Zhao et al. (38) reported that pretreatment with 1 × 10^−5^, 3 × 10^−5^, and 5 × 10^−5^ M of *F* all significantly inhibited the contractile response to KCl in a non-competitive manner. In the current study, the prior exposure to *F* (1 × 10^−6^ M), biochanin A (1 × 10^−6^ M), and their combination did not have an impact on maximum contractile response of mesenteric artery and vein to KCl in treatments either with or without EXT. Since previous studies have indicated that the inhibition effects were dose-dependent (36, 37) and non-competitive (38), it is possible that the isoflavone concentration (1 × 10^−6^ M) in the current study was not high enough to elicit any inhibitory effects on the KCl response. However, in the current study, maximum KCl responses were unaffected by *F* and *B*, which validate the usage of KCl as a reference compound to normalize the contractile responses.

There is limited information about isoflavones’ impact on blood vessel morphology in regards to vessel inside and outside diameters. In the current study, there was no significant evidence of an *F* or *B* effect on mesenteric artery inside or outside diameter regardless of EXT treatment. However, a tendency for a smaller inside diameter induced by *F* treatment was observed in mesentery artery pretreated without EXT. One possible explanation for this could be the inhibitory effect of *F* on vascular smooth muscle cells. Previously, estrogens [tamoxifen (39), estradiol (40)] and isoflavones [*F* and *B* (41)] have been shown to inhibit mitogen-induced proliferation, migration, and extracellular matrix synthesis of smooth muscle cells. On the other hand, the inside diameter of bovine lateral saphenous veins collected from steers grazing high-endophyte tall fescue pasture were smaller than those from steers grazing low-endophyte mixed-grass pasture (16). Likewise, Egert et al. (15) observed a smaller outside diameter of bovine mesenteric artery from endophyte-infected tall fescue seed-dosed steers than control steers. One possible explanation is the prolonged vasoconstriction induced by ergot alkaloids results in decreased inside or outside diameter. Additionally, the morphological changes of blood vessels, especially the expansion of the tunica media smooth muscle layer, could also lead to a smaller vessel insider diameter. It has been observed that calves given ethanolic extracts of tall fescue hay had symptoms of fescue foot and concomitant thickening of vessel walls and smaller vessel lumens in blood vessels of the coronary bands and tail tips (42). Similarly, Garner and Cornell (43) reported a thickening of the smooth muscle layer of peripheral blood vessels after consumption of endophyte-infected tall fescue. Although the exact mechanism associated with thickening of smooth muscle layer is unclear, evidence suggests hyperplasia over hypertrophy. Strickland et al. (44) reported that ergonovine, ergovaline, and α-ergocryptine stimulate the growth and mitosis of quiescent bovine vascular smooth muscle cells *in vitro*. Although, in the present study, there was no significant impact of *F* and *B* pretreatment on mesenteric artery inside or outside diameters, the tendency of larger inside diameter caused by *F* may indicate an alleviative effect on ergot alkaloids induced thickening of vessel walls and smaller lumens.

In the current study for pretreatments without EXT, ERT induced similar contractile response curves and −log^EC50^ values (Control, *F*, *B*, and *F*+*B*) in mesenteric arteries. The shape of contractile responses were similar with Egert et al. (15), who reported ERT-induced contractile responses in mesenteric artery from steers, not exposed to ergot alkaloids, with a −log^EC50^ value of 6.03 ± 0.4 M. Whereas, the shape of mesenteric vein contractile response curves were in contrast to the observations of Egert et al. (15), which did not drop to negative values after reaching a maximum. Further, the maximum contractile response of mesenteric vein to ERT in the current study was also lower (10% vs. 45% of KCl maximum). The blood vessels used in the previous study (15) did not undergo an *in vitro* pre-myograph incubation and utilized blood vessels from steers compared to heifers in the current study.

Substantial evidence has shown that many ergot alkaloids are vasoconstrictive in multiple types of vessels in various animal models (7, 8). Among these vasoactive ergot alkaloids, ERT and ergovaline were indicated as more potent vasoconstrictors with lower EC50 values (relative to other alkaloids) in bovine saphenous vein (45), ruminal vasculature (13), and mesenteric vasculature (15). The current observation of the contractile response induced by ERT was consistent with the previous findings in terms of vasoactivity for this alkaloid. Even though not completely defined yet, numerous studies have been conducted to investigate the mechanism of ergot alkaloid-caused vasoconstriction. The structural similarities of the ergoline ring system and several biogenic amines [i.e., (nor)epinephrine, serotonin, and dopamine] allows ergot alkaloids to interact with corresponding biogenic amine receptors as ligands (30, 46). Substantial evidence has shown that ergot alkaloids interact with dopamine-2 receptors (47, 48), α_1_-adrenergic receptors (49, 50), α_2_-adrenergic receptors (51), and 5-HT_2A_ receptors (14, 16, 17). The binding with these G protein-coupled receptors activates the subunit of the heterotrimeric G protein and then triggers various secondary messaging systems and corresponding cytoplasmic signaling transductions. The vasoconstrictive response induced by ERT shown in the current study (Figure 2) could be explained by these agonistic mechanisms.

The pre-myograph incubation with EXT altered the contractile capacity of the mesenteric artery and vein. Using a bovine lateral saphenous vein model, Klotz et al. (16) found that 2,5-dimethoxy-4-iodoamphetamine (DOI), a 5-HT_2A_ receptor agonist, induced vessel contractile intensities were 35% lower in high endophyte-infested tall fescue than in low-endophyte-infested tall fescue, whereas 5-carboxytryptamine (5-HT_7_ receptor agonist) produced greater (37%) contractile intensities in high endophyte-infected tall fescue. Taken together, this indicated that chronic exposure to ergot alkaloids through grazing endophyte-infested tall fescue altered the vasoconstriction via serotonergic receptors. In a subsequent study, Klotz et al. (17) reported a suppression of the contractile response to ergovaline and 5-HT in steers grazing Kentucky-31 tall fescue infected with wild-type endophyte. Further, Egert et al. (15) demonstrated a similar reduced contractile response to ERT in mesenteric vasculature of steers treated with endophyte-infected tall fescue seed. Unsurprisingly, the antagonistic effects of ergot alkaloids to certain 5-HT receptors have been shown previously (49, 50, 52). Collectively, it is possible that the EXT pretreatment in the present experiment reduced the vasoconstriction to ERT via altering the biogenic amine receptor activities.

Many *in vitro* bioassays have demonstrated that, in various vessel types and in numerous species, the binding of ergovaline and receptor was irreversible, or the dissociation from the receptor was very slow (14, 45, 50, 53). Likewise, an apparent bioaccumulation was reported in bovine lateral saphenous veins after repetitive exposures of ergovaline *in vitro* (54). Additionally, studies have indicated that ergot alkaloid-induced constriction is prolonged and wash-resistant in human superficial temporal artery (55) and coronary artery (56). These findings may explain the high contractile response to the initial ERT addition (5 × 10^−9^ M) in mesenteric arteries that were pretreated with EXT (1 × 10^−6^ M ergovaline) in the current study. Furthermore, using an inositol phosphate accumulation assay, Unett et al. (57) reported that as a 5-HT_2B_ agonist, ERT maintained similar potencies after 2 or 4-h extensive washout and this was due to internalization or sequestration of the active ERT-bound receptor. It has been demonstrated that ERT and ergovaline are equally potent vasoconstrictors inducing similar contractile responses (45). Thus, these findings support the observations in the current study that the elevated contractile response to very low concentrations of ERT pretreated with EXT was numerically similar to 1 × 10^−6^ M ERT-induced contractile response of mesenteric artery without EXT incubation. This can be attributed to prior exposure to ergovaline (in EXT) leading up to the myograph portion of the experiment, and possible carry over of the vascular response to ergovaline to the dose response to ERT.

In the present study, prior exposure to *F* and *B* failed to consistently alter the mesenteric vasculature contraction induced by ERT. As discussed earlier in this section, numerous studies have shown the vasodilative effect of isoflavones and their metabolites in different vessel types. Pretreatment with *F* (3 × 10^−5^ and 5 × 10^−5^ M) antagonized contractile responses of rat thoracic aortas to norepinephrine in a non-competitive manner (38). Sun et al. (37) hypothesized that pre-incubation with *F* (3 × 10^−5^ and 1 × 10^−4^ M) depressed the contraction of rat mesenteric artery to phenylephrine and 5-HT, and similarly, depression was not observed when *F* at 1 × 10^−5^ M. The 1 × 10^−6^ M *F* and *B* concentrations in the present study were possibly not high enough to elicit sufficient vasorelaxation to offset the ERT-induced vasoconstriction. Other evidence of the chronic antihypertensive effect of *F* has been reported based on male spontaneously hypertensive rats (SHR) (58). They found that the vasoconstriction of mesenteric artery segments induced by phenylephrine or 5-HT was reduced in *F* (50 mg/kg per day) orally administrated to SHR. The expression of α_1_-adrenoceptors and 5-HT_2A/1B_ receptors in mesenteric artery of *F* treated SHR decreased (58).

Even though there might be some species differences between bovine and SHR, the 2-h pre-myograph incubation with *F* or *B* in the present study may not be long enough to permit expression or induction of NOS by the tissues that were used. However, sodium- and ATP-dependent K+ transport mechanisms, like those described by Wu et al. (29), are very rapid. Further, *F* and *B* are metabolized to daidzein and genistein in the rumen (59). As many metabolic studies have found, daidzein is further metabolized by rumen and intestinal bacteria to equol, which has a higher estrogenic potency than its isoflavone precursor (22, 23, 59). Equol undergoes renal clearance by clover-fed ruminants (60, 61), which indicates that equol, rather than formononetin, is present in the blood. It is possible that equol and other bacterial metabolites of isoflavones could have greater effects on bovine blood vessels. Thus, the different routes of administration of isoflavone treatments (*in vivo* vs. *in vitro*) may account some of the inconsistencies, and the idea that bacterial or animal metabolism might influence vasorelaxation is consistent with the aforementioned results by Sun et al. (58).

With EXT in the pretreatment media, *F* and *B* increased the contractile response of mesenteric artery at several ERT concentrations. This has provided the first evidence that the isoflavones, *F* and *B*, have potential to alleviate the vasoconstrictive effect of ergot alkaloids. However, based on the current knowledge from ergot alkaloids and isoflavones, there is little to mechanistically explain this phenomenon, so more investigations on their interactions are needed.

## Conclusion

In conclusion, this study indicated that a pre-myograph incubation with *F*, *B* at 1 × 10^−6^ M and their combination did not affect the contractile response to ERT in mesenteric vasculature. The pre-myograph incubation of mesenteric vasculature with EXT (equivalent to 1 × 10^−6^ M ergovaline) reduced the vasoactivity of ERT, and there were some indications that *F* and *B* may alleviate this reduction. At higher concentrations, *F* and *B* may alleviate this reduction in vasoactivity caused by prior exposure to ergot alkaloids. Future studies with higher isoflavone dosages or longer exposure may be helpful to further investigate the ergot alkaloids and isoflavones interaction. Additionally, this study was confined to the mesenteric veins and arteries. It is plausible that effects could be elicited in other vessels with different expression levels of 5-HT, NOS, or other factors. *In vivo* research, which takes isoflavone metabolism into account, is necessary to better understand the interaction from a practical perspective.

## Conflict of Interest Statement

The authors declare that the research was conducted in the absence of any commercial or financial relationships that could be construed as a potential conflict of interest.
